# Nanometer Ammonium Perchlorate and Ammonium Nitrate Prepared with 2D Network Structure via Rapid Freezing Technology

**DOI:** 10.3390/nano9111605

**Published:** 2019-11-12

**Authors:** Yi Wang, Xiaolan Song, Fengsheng Li

**Affiliations:** 1 School of Materials Science and Engineering, North University of China, Taiyuan 030051, China; 2 School of Environment and Safety Engineering, North University of China, Taiyuan 030051, China; 3 School of Chemical Engineering, Nanjing University of Science and Technology, Nanjing 210094, China; lifengsheng424@163.com

**Keywords:** nano AP, nano AN, liquid nitrogen, freeze drying, thermolysis

## Abstract

Nanometer (nano) ammonium perchlorate (AP) and ammonium nitrate (AN) were prepared with 2D network structures by the ultra-low temperature spray method. Scanning electron microscopy (SEM), X-ray diffractometry (XRD), differential scanning calorimetry (DSC) and thermogravimetric analysis/infrared spectrometry (TG-IR) were employed to probe the micron structure, crystal phase, and thermal decomposition of nano AP and nano AN. SEM images revealed that the sizes of nano AP and AN were in the nanometer scale (<100 nm) in one dimension. XRD patterns showed that the crystal phases of nano AP and AN were in accordance with those of raw AP and raw AN, respectively. DSC traces indicated that the thermal decomposition process of AP depended on its particle size, while the thermolysis of AN was independent of the particle size of AN. TG-IR analyses illustrated that the decomposition products of nano AP were NO_2_, N_2_O, HCl and H_2_O, with a small amount of NOCl, and the main decomposition products of nano AN were N_2_O and H_2_O, with a small amount of NH_3_. The results of mechanical sensitivity tests indicated that nano AP was more sensitive than raw AP and both nano AN and raw AN were very insensitive to impact and friction stimuli.

## 1. Introduction

Ammonium perchlorate (AP) and ammonium nitrate (AN) are the most commonly used oxidizers applied in solid propellants [1,2,3]. In addition to their use in propellants, AP and AN are also employed as oxygen-enriched ingredients in explosives and pyrotechnics [4,5,6]. Researchers have found that the performance of AP and AN is a function of their particle sizes. For example, the burning rate of a propellant using superfine AP as an oxidizer is obviously higher than that of the propellant using coarse AP as an oxidizer [7]. However, in contrast to AP, the burning rate of propellants that use AN as an oxidizer is independent of the particle size of AN [8]. Interestingly, a decrease in particle size of AN is beneficial as it decreases the combustion pressure exponent of AN-based propellants [9]. The mechanism concerning this is still obscure, of course, and is also not the theme of this work.

Kumari et al. prepared nanometer (nano) AP by a precipitation method, and the nano AP was precipitated out in the form of nanoparticles in an HTPB (hydroxy terminated polybutadiene) matrix [10]. The HTPB matrix limited the growth of the particles, resulting in a mean size of 33 nm for the nano AP. This method was very efficient because the yield of nano AP was up to 85% (8.5 g per batch). Frolov et al. prepared nano-sized powders of ammonium nitrate through vacuum deposition on a cooled quartz substrate [11]. The AN crystallites were fairly uniform in size and shape and less than 50 nm in diameter. Aside from these two studies, the literature provides a number of other examples on how to prepare superfine AP and AN, which are also of great reference significance. For instance, Ma et al. used acetone as a solvent and ethyl acetate as a non-solvent to prepare superfine AP particles with sizes of 5~10 μm [12]. Wu et al. used an air jet pulverization method to fabricate superfine AP particles with very narrow particle size distribution [13]. Song et al. fabricated spherical superfine AP particles (d_50_ ≈ 5 μm) via two methods [14]. Specifically, coarse AP particles were first comminuted to fine particles (d_50_ ≈ 20 μm) by an air jet pulverization method, and then the fine particles were further pulverized by a mechanical milling method to obtain superfine particles. The high spheroidization degree of such particles resulted in a high density propellant. Compared with coarse AP, the impact sensitivity and friction sensitivity of the superfine AP deceased by 32% and 22%, respectively. Furthermore, differential scanning calorimetry (DSC) indicated that decomposition of the superfine AP occurred earlier than it did for coarse AP, and that the decomposition activation energy of the superfine AP was lower than that of coarse AP. There are few reports on superfine AN. Chai et al. fabricated superfine AN with a particle size of 2~10 μm by a mechanical milling method [15]. In this case, acetone was used as a grinding medium and, after milling, nitrocellulose was coated on the surface of the superfine particle to reduce the hygroscopicity of AN.

The abovementioned researches suggest that mechanical milling may be the best method for fabricating nano AP and nano AN. However, so far, this method has been incapable of pulverizing coarse AP and AN into nanoparticles because the inherent texture of AP and AN crystals determine that they cannot be pulverized to nanoparticles by a mechanical method. In fact, many nano explosives have been fabricated using a mechanical milling method, such as nano RDX [14], nano HMX [16,17], nano CL-20 [18,19], nano PETN [19], nano HNS [20,21], nano TATB [20,22], and nano TAGN [23], with the exception of nano AP and nano AN. Thus, the current study aimed to fabricate AP and AN with nanoscale structures via a liquid nitrogen-assisted and vacuum freeze-drying method.

## 2. Experimental Method

### 2.1. Materials

For this study, ammonium perchlorate (AP) and ammonium nitrate (AN) were purchased from Tianjin Guangfu Chemical Co., Ltd. (Tianjin, China) and liquid nitrogen was purchased from Taiyuan Taineng Gas Co., Ltd. (Taiyuan, China). A mini high pressure atomization pump (ZEKUN, ZK-PW-XT, Shanghai, China), used in the preparation process, was manufactured by Shanghai Zekun Environmental Protection Technology Co., Ltd. (Shanghai, China).

### 2.2. Fabrication of Nano AP and Nano AN

We dissolved 10 g of AP (or 20 g AN) in 190 g (or 180 g for AN) deionized water. The resulting aqueous solution was loaded into the mini high pressure atomization pump. An open container was used to hold about 200 mL of liquid nitrogen. The nozzle of the atomization pump was then aligned with the liquid nitrogen and the pump was started. After all aqueous solutions were sprayed into the liquid nitrogen, the pumped was turned off. As the temperature of the liquid nitrogen was −196 °C and the water and liquid nitrogen were not miscible, all the droplets were frozen to their freezing points instantly. In the preparation process, the liquid nitrogen boiled violently. When all the liquid nitrogen evaporated off, the open container was placed in a freeze dryer and drying commenced. One week later, when all the ice had sublimated, nano AP (or nano AN) was obtained.

### 2.3. Characterization and Tests

Surface morphologies of nano AP and nano AN were investigated using scanning electron microscopy (SEM; JEOL JSM-7500, Tokyo, Japan), while the crystal phases were studied with X-ray diffractometry (XRD; Bruker Advance D8, Karlsruhe, Germany). Further, differential scanning calorimetry (DSC; DSC-100, Nanjing DAZHAN company, Nanjing, China) was employed to investigate the thermal decomposition of samples and thermogravimetric analysis/infrared spectrometry (TG-IR; Mettler Toledo, Zurich, Switzerland) was used to investigate the decomposition products of samples.

The impact sensitivity of the samples was tested with an HGZ-1 impact instrument (North University of China, Taiyuan, China). The special height (*H*_50_) represents the height at which a 5 kg drop-hammer will cause an explosive event in 50% of the trials. In each determination, 25 drop tests were carried out to calculate the *H*_50_. The friction sensitivity of the samples was tested with a WM-1 friction instrument. In each determination, 50 samples were tested, and the explosion probability (P, %) was obtained.

## 3. Results and Discussion

**Figure 1:**
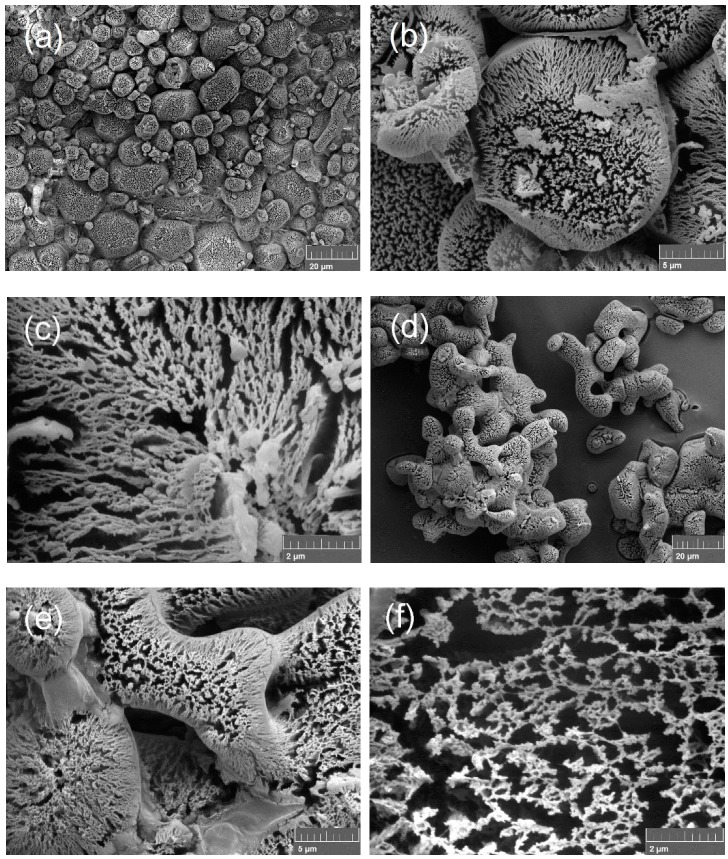
Scanning electron microscopy (SEM) images of samples: (**a**–**c**) nanometer (nano) ammonium perchlorate (AP) and (**d**–**f**) nano ammonium nitrate (AN).

SEM images of nano AP and nano AN are shown in Figure 1. In Figure 1a it is clear that there are many spherical-like “particles” with sizes of 5~10 μm. After increasing the magnification of the image, as shown in Figure 1b, it can be seen that the “particles” are hollow with rough surfaces. After further increasing the magnification of the image, as shown in Figure 1c, it can be observed that the surfaces of the “particles” present a 2D network structure with one-dimensional nanometer size (<100 nm). Figure 1d–f reveals that the micron morphology and structure of nano AN is similar to that of nano AP.

**Figure 2:**
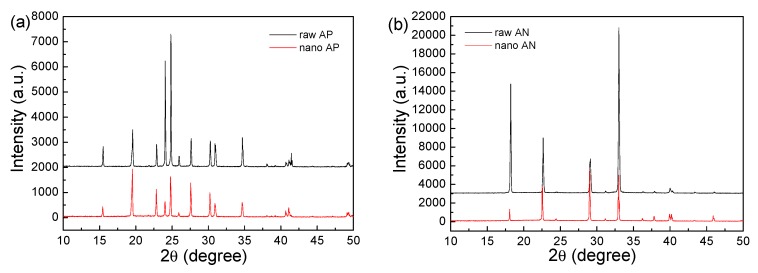
X-ray diffractometry (XRD) patterns of samples: (**a**) raw and nano AP and (**b**) raw and nano AN.

In the fabrication process, the only raw materials were the oxidizer, water, and liquid nitrogen. The water and liquid nitrogen were subsequently evaporated. Thus, it is suggested that no impurity was introduced. However, it was not clear whether the crystal phases of AP and AN transformed in the process. In terms of their practical application, phase transformation is undesired. Consequently, we performed XRD on raw AP, nano AP, raw AN, and nano AN, with the patterns from these analyses illustrated in Figure 2. It is clear that the crystal phases of nano AP and nano AN are consistent with the phases of raw AP and raw AN, respectively. Therefore, we were able to conclude that no phase transformation occurred in the fabrication process.

**Figure 3:**
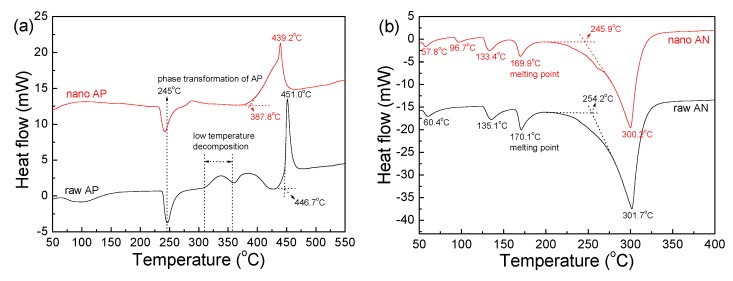
Differential scanning calorimetry (DSC) traces of samples: (**a**) raw and nano AP and (**b**) raw and nano AN.

The DSC traces of raw AP, nano AP, raw AN, and nano AN are shown in Figure 3, produced using a heating rate of 20 °C/min. In Figure 3a, each curve has an endothermic peak at 245 °C, corresponding to the phase transformation of AP. For raw AP, low temperature decomposition occurs in the range of 310–360 °C. However, for nano AP, low temperature decomposition does not occur. Further, the high temperature decomposition of raw AP begins at 446.7 °C and the peak point appears at 451 °C, but the high temperature decomposition of nano AP originates at 387.8 °C and the peak point appears at 439.2 °C. This means that the thermolysis occurred earlier for nano AP than raw AP. It can be seen from Figure 3b that nano AN is quite similar to raw AN in terms of DSC trace. For raw AN, the peaks at 60.4 and 135.1 °C correspond to a phase transformation of AN. The peak at 170.1 °C is related to the melting point of AN. The large endothermic peak at 301.7 °C may be ascribed to the thermal decomposition of raw AN. Upon careful observation, it may be observed that there are four small endothermic peaks in the DSC curve of nano AN. For nano AN, an extra phase transformation occurs at 96.7 °C, which does not exist in the DSC curve of raw AN. The melting point of nano AN is 169.9 °C, which is roughly the same as that of raw AN. The thermal decomposition of nano AN begins at 245.9 °C, slightly lower than raw AN’s 254.2 °C. The peak temperature for thermolysis of nano AN (300.2 °C) is quite close to that of raw AN (301.7 °C), indicating that the decomposition process does not change when the particle size of AN decreases from the micron scale to the nano scale.

With reference to our results, we noted that the thermal decomposition of AN was independent of its particle size, while the thermolysis of AP depended on its particle size. This may be attributed to differences in the decomposition mechanisms of AP and AN. In the first step of their decomposition, AP and AN dissociate to NH_3_ and HClO_4_ (for AP) and NH_3_ and HNO_3_ (for AN) when the temperature reaches a critical point. NH_3_ cannot directly react with HClO_4_ or HNO_3_ at low temperatures. So, further decomposition of HClO_4_ or HNO_3_ is essential for sustained thermolysis of AP or AN. However, the decomposition of HClO_4_ is much easier than that of HNO_3_, as the decomposition process of HClO_4_ is very exothermic and the decomposition process of HNO_3_ is very endothermic [24,25]. Therefore, the rate limiting step of the decomposition of AP lies in the reaction between NH_3_ and the pyrolysis products of HClO_4_, while the controlling step of AN’s decomposition is the pyrolysis of HNO_3_ [26]. In fact, the inferior decomposition mechanism of AN accounts for its remarkably lower burning rate compared to AP. The problem for the decomposition of AP lies in that the NH_3_ and pyrolysis products of HClO_4_ react with each other only on the surface of the decomposing AP particles. The excessive accumulation of NH_3_ gas on the surfaces of AP particles forms a thick ammonia cage, thereby hindering further decomposition reactions [24]. Thus, increasing the specific surface area of AP particles is conducive to alleviating the negative effects of the ammonia cage, therefore promoting the reaction between NH_3_ and the pyrolysis products of HClO_4_. Aside from the case of AP, the reaction between NH_3_ and the pyrolysis products of HNO_3_ is instantaneous, which is partly responsible for the detonable property of AN [27]. Hence, thermolysis of AN does not have problems like the ammonia cage. Please note that AP is not detonable partly because the reaction between NH_3_ and the pyrolysis products of HClO_4_ is not instantaneous. These mechanisms illustrate the discrepancy observed in the DSC results in Figure 3a,b.

**Figure 4:**
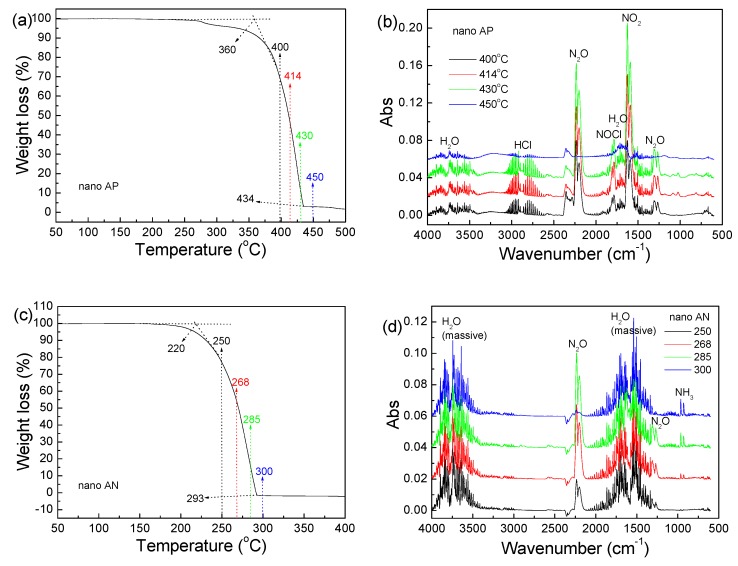
Thermogravimetric analysis/infrared spectrometry (TG-IR) spectra of samples: (**a**,**c**) TG curves and (**b**,**d**) IR spectra for decomposition products of nano AP and nano AN.

To further investigate the thermal decomposition of nano AP and nano AN, TG-IR analysis was conducted at a heating rate of 10 °C/min, the results of which are shown in Figure 4. As observable in Figure 4a, which shows the TG curve of nano AP, decomposition begins at 360 °C and ends at 434 °C. This result is slightly different from the DSC trace (Figure 3a) due to the different heating rates employed in the DSC and TG-IR analyses. The IR spectra at 400, 414, 436, and 450 °C were extracted and are shown in Figure 4b. From the figure, it is obvious that the main gas products of nano AP are NO_2_, N_2_O, HCl, and H_2_O, while some NOCl is also detected. For nano AN, its decomposition products are N_2_O and a massive amount of H_2_O (steam). It should be pointed out that some NH_3_ is produced in the case of nano AN, but it is not present in the decomposition products of nano AP. NH_3_ is produced as a product of the dissociation of AN [28]. In fact, most ammonia is instantaneously oxidized by the NO_2_ produced from the pyrolysis of HNO_3_, which gives rise to the formation of large amounts of N_2_O and H_2_O [29]. Nevertheless, it is hard for NH_3_ to react with N_2_O at low temperatures. So, when the concentration of N_2_O is low, a small amount of NH_3_ remains. In fact, TG-IR analysis of the decomposition of micron AP and AN was conducted in our other papers, and the papers of other authors reported similar results. For example, in Ref. [30], Liu et al. found that micron AP decomposed to NO_2_, N_2_O, HCl, H_2_O, and NOCl, which is in accordance with the results of this work. In Ref. [26], the micron AN decomposed to N_2_O and H_2_O, which is also consistent with the results of this paper. Therefore, on the aspect of final decomposition products, it is believed that the decomposition of AP or AN happens at the molecular level, independent of the particle sizes of AP and AN.

Impact and friction sensitivities of raw AP, nano AP, raw AN, and nano AN were also tested and the results are presented in Table 1. The results show that when the particle size of AP or AN was reduced to the nanometer scale, their sensitivities change significantly. The impact sensitivity of nano AP was slightly higher than that of raw AP, and the friction sensitivity of nano AP was noticeable higher than that of raw AP. However, for AN, the increase in sensitivity was not obvious. The impact sensitivity of nano AN was similar to that of raw AN, and the friction sensitivity of nano AN was somewhat higher than that of raw AN. This is because AN itself is a very insensitive, energetic material. Of course, these results are not consistent with the results reported by Luo or Dobrynin [31,32]. In Luo’s work, the addition of nano LLM-105 was beneficial as it decreased the impact sensitivity of NC/GAP (nitrocellulose/glycidyl azide polymer) fibers, and in Dobrynin’s work nano NC presented lower friction sensitivity than micron NC. Now, it cannot yet be elucidated why nano AP is more sensitive than raw AP, but nano NC is less sensitive than raw NC. However, at this time, this discrepancy may simply be attributed to the different decomposition mechanisms of AP and NC (NC is nitrocellulose as a nitrate ester) [33]. The decomposition of AP begins when it dissociates to NH_3_ and HClO_4_, while decomposition of NC originates from the rupture of O–NO_2_ bonds. Further research on this topic will be continued in our next work.

## 4. Conclusions

Nano AP and nano AN, with structures that were in the nanometer scale in one dimension, were prepared with an ultra-low temperature spray method. The micron morphologies of nano AP and nano AN were 2D network structures. XRD analysis confirmed that crystal phase transformation did not occur in the fabrication process. For DSC curves, compared with raw AP, the peak temperature of nano AP was larger by 12 °C. The thermal decomposition of AN was found to be independent of its particle size; that is, the DSC peak point of nano AN was roughly the same as that of raw AN. The decomposition products of nano AP were NO_2_, N_2_O, HCl, and H_2_O, as well as a small amount of NOCl, while the main decomposition products of nano AN were N_2_O and H_2_O, with a small amount of NH_3_. The impact sensitivity of nano AP was somewhat higher than that of raw AP and the friction sensitivity of nano AP was noticeably higher than that of raw AP. But, in the case of AN, the increase of sensitivity was not obvious.

## Figures and Tables

**Table 1:** Impact and friction sensitivities of samples.

Samples	Impact Sensitivity (H_50_, cm)	Friction Sensitivity (P, %)
raw AP	95	26
nano AP	83	94
raw AN	>120	0
nano AN	>120	8
